# Antimicrobial Susceptibility of *Enterococcus* Isolates from Cattle and Pigs in Portugal: Linezolid Resistance Genes *optrA* and *poxtA*

**DOI:** 10.3390/antibiotics11050615

**Published:** 2022-05-03

**Authors:** Joana Gião, Célia Leão, Teresa Albuquerque, Lurdes Clemente, Ana Amaro

**Affiliations:** 1 Laboratory of Bacteriology and Mycology, INIAV—National Institute of Agrarian and Veterinary Research, 2780-157 Oeiras, Portugal; joanagiao.silva@outlook.pt (J.G.); celia.leao@iniav.pt (C.L.); teresa.albuquerque@iniav.pt (T.A.); lurdes.clemente@iniav.pt (L.C.); 2 Department of Veterinary Medicine, University of Évora, 7002-554 Évora, Portugal; 3 MED—Mediterranean Institute for Agriculture, Environment and Development, 7006-554 Évora, Portugal; 4 CIISA—Centre for Interdisciplinary Research in Animal Health, Faculty of Veterinary Science, University of Lisbon, 1300-477 Lisboa, Portugal

**Keywords:** *Enterococcus* spp., pigs, cattle, Linezolid resistance, *optrA* gene, WGS

## Abstract

Enterococci are part of the commensal gut microbiota of mammals, with *Enterococcus faecalis* and *Enterococcus faecium* being the most clinically relevant species. This study assesses the prevalence and diversity of enterococcal species in cattle (*n* = 201) and pig (*n* = 249) cecal samples collected in 2017. Antimicrobial susceptibility profiles of *E. faecium* (*n* = 48) and *E. faecalis* (*n* = 84) were assessed by agar and microdilution methods. Resistance genes were screened through PCR and nine strains were analyzed by Whole Genome Sequencing. A wide range of enterococci species was found colonizing the intestines of pigs and cattle. Overall, the prevalence of resistance to critically important antibiotics was low (except for erythromycin), and no glycopeptide-resistant isolates were identified. Two daptomycin-resistant *E. faecalis* ST58 and ST93 were found. Linezolid-resistant strains of *E. faecalis* (*n* = 3) and *E. faecium* (*n* = 1) were detected. Moreover, oxazolidinone resistance determinants *optrA* (*n* = 8) and *poxtA* (*n* = 2) were found in *E. faecalis* (ST16, ST58, ST207, ST474, ST1178) and *E. faecium* (ST22, ST2138). Multiple variants of *optrA* were found in different genetic contexts, either in the chromosome or plasmids. We highlight the importance of animals as reservoirs of resistance genes to critically important antibiotics.

## 1. Introduction

The *Enterococcus* genus comprises over 50 species of ubiquitous Gram-positive bacteria found in the environment and the gastrointestinal tract of various hosts, including humans and other mammals, birds, and invertebrates, as part of their normal microbiome [1,2]. The presence and diversity of enterococci species can significantly vary according to host species, age, diet, gastrointestinal tract region, environmental stress, and season [3,4,5]. *Enterococcus faecalis* and *Enterococcus faecium* represent up to 1% of the adult gut microbiota in humans [4] and are the two most relevant species associated with multidrug resistance and nosocomial infections.

Although members of the *Enterococcus* genus are considered commensal bacteria, they can also become opportunistic pathogens in favorable environmental conditions. Hospital-acquired enterococcal infections became a cause of global concern due to their increasing prevalence and resistance to several classes of antibiotics [6]. These bacteria are also known for efficiently recruiting and exchanging antibiotic resistance determinants. Various enterococci strains acquired resistance to many last-resort antibiotics, such as vancomycin, daptomycin, linezolid and tigecycline [7]. Glycopeptides, lipopeptides, oxazolidinones and glycylcyclines have been placed in category A (Avoid) of the European Medicines Agency (EMA) categorization of antibiotics in the European Union (EU), currently not being approved for veterinary use [8].

Studies suggest that *E. faecium* isolates from animals may act as donors of antibiotic resistance determinants to human-adapted bacteria after ingestion of products of animal origin [9]. There is also evidence that *E. faecalis* strains from animals may be considered a hazard to humans [9]. For instance, the emergence of vancomycin-resistant enterococci (VRE) due to the overuse of the vancomycin analog avoparcin as a growth promotor in farm animals is suspected of having contributed to human VRE outbreaks in some countries [10].

Under the One Health concept, EU countries have implemented surveillance programs monitoring resistance to critically important antibiotics of commensal bacteria from farm animals [11]. In the case of enterococci, these antibiotics include last-resort antibiotics such as glycopeptides, linezolid, and daptomycin. Nevertheless, enterococci surveillance is not mandatory, and thus in several countries, no antimicrobial resistance surveillance program is routinely applied to *Enterococcus* spp. in farm animals yearly, including Portugal.

Resistance to glycopeptides in enterococci has been mostly associated with the *vanA* and *vanB* gene clusters that allow for the synthesis of alternative cell wall precursors with low binding affinity to vancomycin [12]. Linezolid resistance can be linked with mutations in the V domain of the 23S rRNA and the *rplC/rplD* genes coding for the L3/L4 ribosomal proteins or with the acquisition of oxazolidinone resistance genes such as *cfr* [13], which encodes a 23S rRNA modifying methyltransferase, and *optrA* [14] and *poxtA* [15], two genes encoding ABC-F proteins that presumably protect the ribosomal target from binding to the antibiotic. The underlying mechanisms conferring reduced susceptibility in enterococci are not entirely understood regarding daptomycin. Non-susceptibility to daptomycin has been connected to mutations in multiple genes. Most of them involved cell envelope stress response, metabolism of important cell membrane phospholipids, or peptidoglycan biosynthesis [7,16].

The usage of glycopeptides in farm animals has been unauthorized in the EU for over two decades. However, vancomycin-resistant enterococci carrying the *van* operon (particularly the *vanA* gene) were still being recovered from samples of food-producing animals years after the ban [9,17,18,19,20].

Resistance to the last-resort antibiotics linezolid and daptomycin has been reported in enterococci strains from European countries including Portugal [7]. In 2018, the overall prevalence of resistance to last-resort antibiotics among enterococci from humans in European countries (such as Denmark, Poland, Spain, Ireland, France, and Portugal) was very low (1%) [7]. Because those antibiotics are not approved for veterinary use in the EU, reports of resistant strains are more frequent in humans. Nevertheless, linezolid resistant enterococci from food-producing animals have been described in Europe [21,22,23]. Very few linezolid resistant *E. faecium* and *E. faecalis* isolates from broilers, cattle, and pigs were detected in countries such as Belgium, Croatia, France, Spain, and The Netherlands [22]. *Enterococcus* carrying the *poxtA* and *optrA* genes have been found in samples from a swine farm in Spain [24] and various food-producing animals in Belgium [23]. An emergence of linezolid-resistant human clinical *Enterococcus* isolates harboring these genes was also registered in European countries [25,26,27,28].

Regarding daptomycin, non-susceptibility is rare in humans and can emerge with or without prior exposure to the antibiotic [29]. Daptomycin non-susceptible enterococci have been reported in samples of food-producing animals from Lithuania and Denmark [30,31].

Here, we aim to investigate the diversity and frequency of gut colonization of cattle and pigs by *Enterococcus* and evaluate the antimicrobial susceptibility patterns of *E. faecalis* and *E. faecium* strains. Moreover, some antimicrobial resistance determinants to critically important antibiotics, including vancomycin and linezolid were also searched. To our knowledge, this study reports, for the first time in Portugal, the occurrence of daptomycin non-susceptible enterococci and the linezolid resistance-encoding genes *optrA* and *poxtA* from food-producing animals.

## 2. Results

### 2.1. Enterococcus Isolation and Species Diversity

A total of 314 presumptive *Enterococcus* spp. were isolated among 450 bovine and swine cecal samples. The genus-specific PCR assay confirmed that 292 isolates belonged to the *Enterococcus* genus, 138 were recovered from cattle and 154 from pigs, with recovery rates averaging around 69% for bovine and 62% for swine samples.

**Figure 1:**
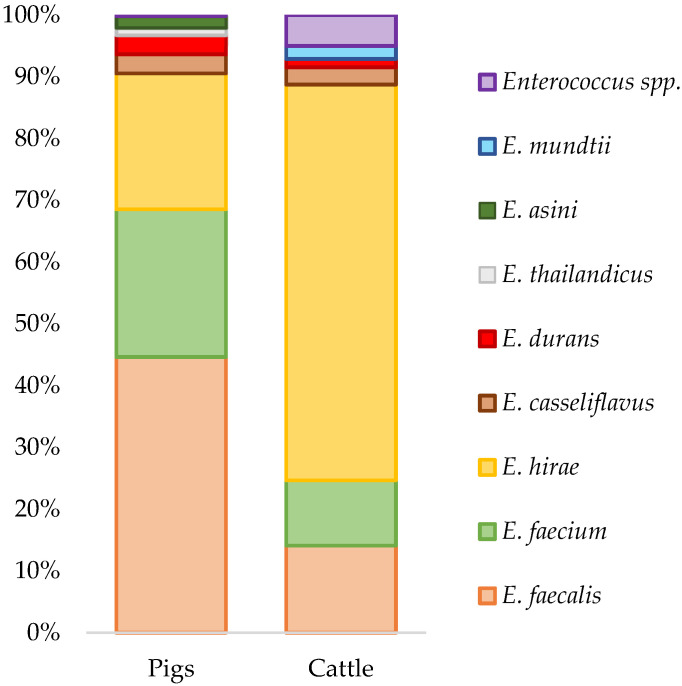
Distribution of *Enterococcus* species in cattle and pigs.

The distribution and diversity of *Enterococcus* species identified from the cecal samples of healthy bovines and swine are illustrated in Figure 1. The isolates without identification to the species level (*n* = 13) remained classified as *Enterococcus* spp.

Among the 292 *Enterococcus* spp. strains, the multiplex PCR assays identified 85.6% of the isolates as *Enterococcus hirae* (*n* = 107), *Enterococcus faecalis* (*n* = 84), *Enterococcus faecium* (*n* = 48), *Enterococcus casseliflavus* (*n* = 7) and *Enterococcus durans* (*n* = 4). Sanger sequencing of the 16S rRNA gene allowed the identification of *Enterococcus hirae* (*n* = 6), *Enterococcus asini* (*n* = 3) and *Enterococcus thailandicus* (*n* = 2). Additionally, the API^®^ 20 Strep system also identified *E. hirae* (*n* = 12), *E. casseliflavus* (*n* = 1) and *E. durans* (*n* = 2).

The most frequent species of *Enterococcus* found in pigs were *E. faecalis* (42.9%), *E. faecium* (23.4%), and *E. hirae* (22.8%), representing nearly 90% of the isolates (Figure 1). Other species of *Enterococcus*, namely *E. durans* (*n* = 4), *E. casseliflavus* (*n* = 4), *E. thailandicus* (*n* = 2), and *E. asini* (*n* = 3), were also found. *E. hirae* was the most abundant species recovered from bovine cecal samples, comprising 65.2% of the isolates (Figure 1). Other species recovered from cattle included *E. faecalis* (*n* = 18), *E. faecium* (*n* = 12), *E. casseliflavus* (*n* = 4), *E. durans* (*n* = 2) and *E. mundtii* (*n* = 3).

### 2.2. Antimicrobial Susceptibility Testing by Agar Dilution

The antimicrobial susceptibility profiles of 84 *E. faecalis* and 48 *E. faecium* isolates from cattle (*n* = 30) and pigs (*n* = 102) were established. Important parameters comprising the MICs_50_, MICs_90_ and the frequencies of decreased susceptibility are summarized in Table 1.

Overall, this study showed moderate to high decreased susceptibility rates to tetracycline (44–98%). Isolates with decreased susceptibility to erythromycin were significantly more prevalent in pigs (*p*-value ≤ 0.05) for both *E. faecium* and *E. faecalis* strains.

No isolates were resistant to glycopeptides, namely vancomycin and teicoplanin. Regarding *E. faecium*, decreased susceptibility to erythromycin was high (58%) and found exclusively in isolates from pigs. Decreased susceptibility to chloramphenicol and ampicillin was rarely observed in isolates from both animal species. Moreover, no isolates displayed decreased susceptibility to ciprofloxacin, gentamicin, and linezolid.

Concerning *E. faecalis* isolates, decreased susceptibility to chloramphenicol and tetracycline was prevalent in pigs (*p*-value ≤ 0.05), followed by erythromycin (17–86%). Among seven isolates of non-wild-type gentamicin, six were also resistant to ciprofloxacin. One *E. faecalis* isolated from pigs displayed resistance to linezolid (MIC = 8 µg/mL). Resistance to ampicillin was not found.

Major differences (over three dilution steps) between MIC_50_ and MIC_90_ values were found for tetracycline in *E. faecium* isolates of bovine origin, gentamicin in *E. faecalis* isolates from pigs, and erythromycin in *E. faecalis* from cattle.

**Figure 2:**
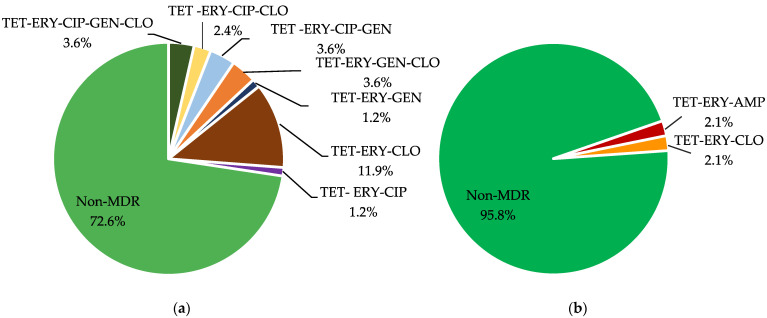
Diversity of multidrug resistance profiles found in (**a**) *E. faecalis* (*n* = 84) and (**b**) *E. faecium* (*n* = 48) strains from swine using the agar dilution susceptibility test. MDR, Multidrug-resistant; TET, Tetracycline; ERY, Erythromycin; LZD, Linezolid; AMP, Ampicillin; CLO, Chloramphenicol; CIP, Ciprofloxacin; GEN, Gentamicin.

Multidrug resistance (MDR) was noticed in 27.4% of *E. faecalis* and 4.2% of *E. faecium* isolates from pigs (Figure 2). The most prevalent MDR profile in *E. faecalis* strains was tetracycline-erythromycin-chloramphenicol. Two different multidrug resistance profiles were found among *E. faecium* strains, one of them unique to this species. Seven MDR patterns were identified in *E. faecalis* isolates. Resistance to both tetracycline and erythromycin was present in all. Full susceptibility was observed in 27.0% of *E. faecium* and 11.9% of *E. faecalis* and more frequent in *E. faecalis* isolates from bovines than swine (*p*-value ≤ 0.05).

### 2.3. PCR Screening of Antimicrobial Resistance Determinants

Ninety isolates with linezolid MIC values of 1–8 μg/mL were subjected to the PCR assays targeting the *optrA* gene. The *optrA* gene was detected in six strains of *E. faecalis* and two *E. faecium*, all sourced from pigs.

Among the isolates exhibiting decreased susceptibility to chloramphenicol (*n* = 20), none harbored the *cfr* gene.

All isolates were negative for the detection of *vanA* and *vanB* genes, corroborating the vancomycin susceptibility profile observed.

### 2.4. Antimicrobial Susceptibility Testing of optrA Positive Strains by Microdilution

**Figure 3:**
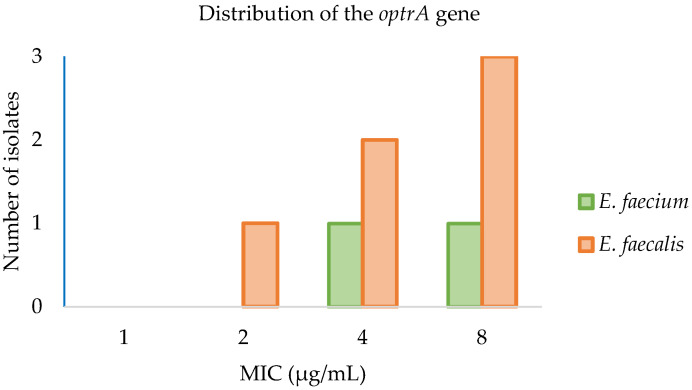
Distribution of linezolid MIC values among strains of *E. faecalis* (*n* = 6) and *E. faecium* (*n* = 2) carrying the *optrA* gene using the EUVENC microdilution plates.

All isolates harboring the *optrA* gene (*n* = 8) were further subjected to antimicrobial susceptibility testing using commercially available EUVENC microplates to confirm linezolid MIC values obtained by agar dilution (Figure 3). An additional set of 13 isolates (nine *E. faecalis* and four *E. faecium*) was also tested.

Overall, the results obtained using the EUVENC microplates were like those obtained by the agar dilution technique (data not shown). For some antibiotics, such as linezolid and chloramphenicol, a few strains exhibited MICs one dilution step higher in the EUVENC microplates compared to the agar dilution technique.

Notably, three isolates (*E. faecium* INIAV004, *E. faecalis* INIAV168 and *E. faecalis* INIAV171) displayed linezolid MIC = 4 µg/mL using the agar dilution technique and MIC = 8 µg/mL in the EUVENC plates. All isolates carrying the *optrA* gene displayed decreased susceptibility to chloramphenicol (MIC > 32 µg/mL) in the EUVENC microplates.

Two *E. faecalis* isolates from pigs (INIAV005 and INIAV175) showed decreased susceptibility to daptomycin with MICs = 8 µg/mL and 16 µg/mL, respectively.

### 2.5. Genomic Characterization of Isolates

The genomic content of nine strains, including MLSTs, acquired resistance genes, and virulence genes are shown in Appendix A Table A1.

The strains belonged to eight different sequence types, including ST16, ST58, ST93, ST207, ST474 and ST1178 for *E. faecalis* and ST22 for *E. faecium* INIAV004. In addition, a novel *E. faecium* sequence type was observed in isolate INIAV173, submitted to PubMLST and assigned as ST2138.

A great variety of virulence factors were found in all strains of *E. faecalis*, which included the *elrA*, *srtA*, *ace*, *agg*, *cCF10*, *cOB1*, *cad*, *camE*, *cylA*, *cylL*, *cylM*, *ebpA*, *ebpC*, *efaAfs*, *hylA* and *tpx* genes; *acm* and *efaAfm* virulence genes were detected in *E. faecium* strains.

Other than INIAV005, all isolates carried *repUS43.* Other plasmid replicons found were *repUS1*, *rep9a*, *rep9b* and *rep6* in *E. faecalis* strains and in *rep1*, *rep2*, *rep11c*, *rep18b*, *rep29*, *repUS15* and *rep33* in *E. faecium* strains.

The following antimicrobial resistance genes were detected: *erm*(A) and *erm*(B) (conferring the macrolide-lincosamide-streptogramin B resistance profile), *ant(9)-Ia*, *aac(6*′*)-aph(2*″**) and *aph(3*′*)-III* (aminoglycoside resistance genes), *tet*(M) and *tet*(L) (encoding resistance to tetracyclines), *dfrG* (a trimethoprim resistant determinant), *lnu*(B) and *lsa*(E) (which confer the lincosamide and pleuromutilin-lincosamide-streptogramin A phenotypes, respectively), *caA1tA*, *catB*, *fexA* and *fexB* (encoding phenicol resistance), *poxtA* (conferring decreased susceptibility to phenicols, oxazolidinones and tetracyclines) and *optrA* (a gene that can confer resistance to oxazolidinones and phenicols).

Genomic analyses of the *E. faecium* isolate INIAV004 also revealed the presence of several mutations of the *pbp5* gene encoding a low-affinity species-specific class B penicillin-binding protein PBP5. *Enterococcus faecalis* INIAV169, INIAV170 and INIAV174 showed mutations in the *gyrA* and *parC genes*, both encoding resistance to quinolones.

#### 2.5.1. Molecular Characterization of Linezolid Resistance Mechanisms

The *optrA*-harboring strains, variants [31,32,33], and linezolid MICs are mentioned in Table 2.

A total of four different OptrA variants were identified: WT, DP, EDD, and DVD.

The isolates carrying *optrA* variants EDD (*E. faecalis* strains INIAV169, INIAV170 and INIAV174) and DVD (*E. faecium* INIAV173) displayed linezolid MICs ≤ 4 µg/mL, while the strains with the WT (*E. faecium* INIAV004) and DP (*E. faecalis* strains INIAV168 and INIAV171) variants consistently exhibited linezolid MICs > 4 µg/mL.

Both *Enterococcus faecium* strains INIAV004 and INIAV173 also harbored the *poxtA* gene with 100% nucleotide sequence identity with the wild-type gene (GenBank accession number MH746818.1). In these two isolates, the *poxtA* and *optrA* genes were in separate contigs.

In isolates with decreased susceptibility to linezolid, mutations in the 23S rRNA were not detected and mutations leading to amino acid changes in proteins L3 and L4 were also not identified through sequence alignment.

#### 2.5.2. Genetic Environment of the *optrA* Gene

Analyses of the contigs containing the *optrA* gene in isolates INIAV169, INIAV170, INIAV174, and INIAV173 revealed that in these strains the gene appeared to be in the chromosomal DNA. In the case of isolates INIAV004, INIAV168, INIAV171, the *optrA* gene was seemingly located in plasmids.

In all genetic backgrounds surrounding the *optrA* gene, the *ermA* or an *ermA*-like gene was present. In the case of *E. faecalis* isolates INIAV168 and INIAV171, the *ermA*-like gene was detected downstream of *optrA* while in all other isolates the *ermA* gene was located upstream of *optrA*. The *fexA* gene was also present upstream of *optrA* in *E. faecalis* strains INIAV168, INIAV169, INIAV170 and INIAV171.

**Figure 4:**
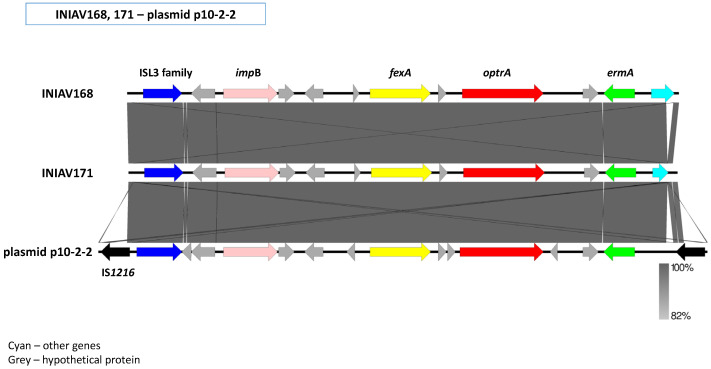
Alignment of the *optrA*-containing contigs of strains INIAV168 and INIAV171 with the previously described *optrA*-carrying plasmid p10-2-2 (GenBank accession number KT862775).

The genetic context of the *optrA* gene was similar in *E. faecalis* strains INIAV168 and INIAV171. The *impB*, *fexA*, and *ermA* genes surrounded *optrA* in an *impB*-*fexA*-*optrA*-*ermA* arrangement flanked upstream by an ISL3-like element. The respective contigs had nucleotide sequences with 100% identity (query cover 100%) with the previously described plasmid p10-2-2A (GenBank accession number KT862775). These sequences were aligned and are presented in Figure 4.

In the isolates carrying the *optrA* gene in the chromosome, no transposable elements were found in the vicinity of the gene.

## 3. Discussion

In this study, we analyzed isolates from the caeca of cattle and pigs collected in 2017 under the scope of the surveillance program of antimicrobial resistance in zoonotic and commensal bacteria. Several species of enterococci were found colonizing the intestine of cattle and pigs, such as *E. faecalis*, *E. hirae*, *E. faecium*, *E. durans*, and *E. casseliflavus.*

The predominant species found in cattle was *E. hirae* and in pigs were *E. faecalis*, *E. hirae*, and *E. faecium*. Our results are similar to those found in other studies [35,36,37,38,39,40], although the relative frequencies of each *Enterococcus* species may vary between studies due to differences in diet, host, and environment-associated factors. Species identification by Sanger sequencing allowed the identification of *E. asini* and *E. thailandicus* in swine, two species seldomly reported in pigs [40], and *E. mundtii*, which is common in cattle [37]. However, thirteen isolates remained as *Enterococcus* spp., either because the species were not included in our PCR assays or due to the low discriminatory power of the 16S rRNA gene when differentiating closely related enterococcal species. Therefore, other techniques such as matrix-assisted laser desorption ionization time-of-flight mass spectrometry (MALDI-TOF MS) or Sanger sequencing of additional genes such as the *sodA* or *tuf* genes could be applied [41].

Overall, the results of antibiotic susceptibility testing obtained are comparable to those found in a previous study assessing the antibiotic susceptibility of enterococci from healthy food-producing animals collected in different European countries from 2004 to 2014 [21].

The frequency of antimicrobial decreased susceptibility was higher for several antibiotics in *E. faecalis* compared with *E. faecium* strains, particularly in swine (*p*-value ≤ 0.05). Decreased susceptibility to tetracyclines was widespread. Except for erythromycin, the prevalence of decreased susceptibility to glycopeptides and oxazolidinones, both critically important antibiotics in human medicine, was either low or absent. These results are in accordance with reports showing tetracyclines and macrolides among the most purchased antibiotic classes in Portugal between 2010 and 2018 (ESVAC) [42].

Decreased susceptibility to ampicillin was displayed only by *E. faecium* isolates from swine, as expected since ampicillin is very rare in *E. faecalis* strains. Decreased susceptibility to ampicillin and gentamicin was not observed in any animal species. These antibiotics are frequently used in combination to treat enterococcal infections, and co-resistance to both antimicrobials is uncommon [21].

Regarding gentamicin, MIC_50_ and MIC_90_ values observed in *E. faecalis* isolates from pigs may suggest the presence of more than one subpopulation. However, large MIC_50_ and MIC_90_ differences between isolates from cattle may not indicate the same since only a few isolates (under 18) were studied [43].

Although decreased susceptibility to chloramphenicol was found in isolates from both *Enterococcus* species, the *cfr* determinant was not detected among these strains, indicating that other phenicol resistance determinants may be present.

In the present study, phenotypic and genotypic resistance to glycopeptides was not observed. Our results contrast with a previous study reporting the *vanA* operon in isolates from 2005 to 2012 collected from food-producing animals in Portugal [44]. Therefore, the absence of vancomycin resistance determinants seen in our isolates is most likely due to the ban on glycopeptides usage in food-producing animals in 1997 [45]. These results are encouraging because they may indicate that this resistance mechanism was finally eradicated in cattle and pigs from Portugal 20 years after banning avoparcin. Nevertheless, national surveillance programs also focused on *Enterococcus* spp. should be implemented on farm animals to confirm these results.

In the present study, the frequencies of MDR in enterococci from farm animals were similar to those described in other European countries [31,46] but lower than those reported in the United States [47], China [48], and Malaysia [49]. MDR isolates were exclusively sourced from swine, while isolates with susceptibility to all antibiotic classes were found more frequently in cattle. The differences between animal species regarding the levels of decreased antibiotic susceptibility and prevalence of MDR strains could reflect the distinct husbandry and antibiotic use practices employed in cattle and pig farming in Portugal.

Regarding antibiotic use for animals in Portugal, overall sales fluctuated, showing a peak in 2016, followed by a decrease in 2017 [42]. In 2020, an overall 19.9% increase in sales was recorded compared to 2019, with tetracyclines, penicillins and macrolides continuing to be the most frequently purchased classes of antibiotics [42].

Globally, we found three *E. faecalis* and one *E. faecium* clinically resistant to linezolid after using the EUVENC microplates. Three *optrA*-carrying enterococci susceptible to linezolid in the agar dilution test were clinically resistant to linezolid (MIC > 4 µg/mL) using the microdilution technique. The one-fold discrepancy observed between methods is common and may occur using different methods due to inherent methodology variations [50].

Antimicrobial resistance profiles predicted by WGS were generally consistent with those displayed in the antibiotic susceptibility tests performed. Interestingly, *E. faecalis* INIAV171 harbored the gene encoding the bifunctional aminoglycoside modifying enzyme AAC(6′)-Ie/APH(2′)-Ia, which confers resistance to a broad spectrum of aminoglycosides, including high-level gentamicin resistance [51,52], but was susceptible to this antimicrobial agent (MIC = 64 μg/mL). *Enterococcus faecium* INIAV004 was predicted to be resistant to ampicillin but remained susceptible to this antibiotic despite showing several *pbp5* point mutations. This may happen due to the absence of specific amino acid substitutions, such as M485A and E629V, occurring mostly around the active-site region of PBP5 or mutations associated with the addition of a serine at position 466, which are more often responsible for increased MICs to ampicillin in enterococci [53,54,55]. Other factors, such as regulation, expression, and translational modifications of the *pbp5* gene or other genes, can also interfere with ampicillin MICs [56].

Oxazolidinone resistance determinants *optrA* and *poxtA* were found in the present study in enterococci isolates from healthy pigs. These results are most likely associated with the extensive veterinary use of other antimicrobials such as florfenicol and tiamulin [57] since oxazolidines are not approved for veterinary use on farm animals [8,58]. The *optrA* gene was identified in eight isolates and the presence of this gene did not always confer clinical resistance to linezolid. In addition, the *poxtA* gene (known to confer decreased susceptibility to phenicols, oxazolidinones, and tetracyclines) was co-carried in *E. faecium* strains INIAV004 and INIAV173, but INIAV173 remained susceptible to linezolid. Linezolid resistant isolates did not possess the *cfr* gene or additional mutations of the *rplC*, *rplD* and 23S rRNA genes.

Isolates carrying the *optrA* gene all belonged to different sequence types, except for *E. faecalis* strains INIAV169 and INIAV170, which belonged to ST474. *E. faecalis* ST474 and ST207 (assigned to INIAV168) carrying the *optrA* gene have already been found in human clinical isolates [59,60,61].

Of all *E. faecalis* sequence types, ST16 is the most frequently associated with the *optrA* gene and has been recovered from food-producing animals and several human clinical samples from various countries, [33,60,62,63,64,65,66]. Linezolid resistant *E. faecalis* INIAV171 ST16 strain is MDR co-harboring acquired antimicrobial resistance determinants associated with resistance to several antibiotic classes, including phenicols, tetracyclines, macrolides, aminoglycosides, lincosamides, pleuromutilins, and trimethoprim. ST16 is considered a zoonotic lineage involved in antimicrobial resistance dissemination [67].

The *E. faecium* INIAV004 strain belonged to ST22 and carried both the *poxtA* and *optrA* genes. Previous studies have reported ST22 strains co-carrying the *optrA* and *poxtA* genes sourced from human patients, poultry, swine, and bovines [7,23,68].

Nomenclature for OptrA variants is not uniform among different studies [32,33,34] creating some difficulties in comparing studies, and thus an extra effort should be made to standardize the designations of these variants.

In this report, strains with the same OptrA variants displayed similar susceptibility to linezolid. MIC values associated with each variant were close to or within the ranges that have been previously reported [32,34,66,69]. Although the same OptrA variants have displayed differences in linezolid MICs across studies, mutations of the *optrA* gene still appear to influence linezolid resistance levels [66,69]. In enterococci carrying *optrA*, linezolid MICs also seem to correlate with the genetic context surrounding the gene [69].

The *optrA* gene appeared to be carried either in the chromosome or in plasmids and with different genetic environments in the analyzed isolates. We often found *fexA* and *ermA* (or *ermA*-like genes) were located close to the *optrA* gene, as observed in many isolates with multiple genetic contexts [28,33,69,70]. Both *E. faecalis* strains INIAV168 and INIAV171 harbored the DP OptrA variant and shared an identical ISL3-*impB*-*fexA*-*optrA*-*ermA* genetic arrangement. The contigs containing these genes were highly similar to plasmid p10-2-2, which has also been described in the *E. faecalis* strain 10-2-2 (GenBank accession no KT862775) sourced from swine. Due to the short sequence size of the contigs, we were not able to determine the presence of the two IS*1216* elements bracketing the *optrA*-carrying central region in p10-2-2, which could allow the mobilization of this DNA region [70].

In enterococci isolates harboring the *optrA* gene in the chromosome, the *radC* gene has often been reported as a favored site for the insertion of Tn554 family-flanked segments containing the *optrA* gene, disrupting the *radC* gene [28,33,34,69,70]. However, we did not find transposons from the Tn554 family (such as Tn558 and Tn6674), nor the associated *tnpA*, *tnpB*, and *tnpC* transposase genes or the *radC* gene in the vicinity of the *optrA* gene in isolates carrying this gene in the chromosome. In these strains, no other transposable elements were identified surrounding the *optrA* gene.

The putative transcriptional regulator *araC* gene frequently found in the upstream region of *optrA* was also not detected in any isolate.

The two daptomycin non-susceptible *E. faecalis* strains from swine reported in our study were susceptible to other antibiotics except for erythromycin and tetracycline. The daptomycin non-susceptible *E. faecalis* INIAV005 belonged to ST58, a sequence type mainly found in pigs [71,72] and *E. faecalis* INIAV005 belonged to ST93, which has been detected in multiple animal species [73,74] and in humans [75,76,77].

There are no daptomycin formulations approved for animal use in the EU, and cross-resistance between daptomycin and other veterinary-approved antibiotic classes has not been reported. Although non-susceptibility to daptomycin has been associated with exposure to the antibiotic, the development of non-susceptibility without prior daptomycin use has also been documented [29]. Thus, daptomycin non-susceptible enterococci emerged most likely due to spontaneous mutations. Nevertheless, the inappropriate use of this drug and the transmission of daptomycin non-susceptible enterococci from humans cannot be dismissed. The molecular basis associated with daptomycin non-susceptibility in these strains has not been clarified yet, and further investigation should be carried out.

*Enterococcus* spp. are frequently considered food contaminants, although the risk of transmission from animals to humans through the food chain is based on indirect evidence [67,78]. The food and animal industries seem to have contributed to the spread of multidrug-resistant strains and certain lineages like *E. faecalis* ST16, considered a zoonotic pathogen [67]. In our study, we identified resistance genes (e.g., *optrA*, *poxtA*, *fexA*, *ermA*) [28,33,69,70], ISs (e.g., IS*1216*) [68], and STs (e.g., ST16, ST22) [26,67] like those observed in humans and other animal species. These findings highlight the risk of the spread of antimicrobial resistance between animals and humans in the farm, slaughterhouse, and retail store environments [8]. Moreover, *optrA* genes were found to be co-located with phenicol resistance gene *fexA* and macrolide resistance gene *ermA*, and thus amphenicol use (or macrolide) could also result in cross-selection of linezolid resistant gene *optrA* (and possibly *poxtA*). It is important to stress that increased amphenicols sales have been observed from 2011 to 2020 in European countries, including Portugal [42]. Amphenicols are currently listed as highly important antimicrobials for humans, placed in category C (Caution) of EMA’s categorization of antibiotics in the EU [8]. Nonetheless, their rational use in veterinary settings should be emphasized to prevent the potential spread of resistance.

Our findings underline the risk of frequent and independent acquisition and selection events for antimicrobial resistance on farms through the pressure of antimicrobials usage in animal production.

Only a collaborative, multisectoral and transdisciplinary approach working at the local, regional, national, and global levels can achieve better public health, recognizing the interconnection between people, animals, and their shared environment.

## 4. Materials and Methods

### 4.1. Bacterial Isolation and Species Identification

Under the scope of the surveillance program of antimicrobial resistance in zoonotic and commensal bacteria (Commission Decision 2013/652/EU), cecal samples from randomly selected healthy bovines (*n* = 201) and swine (*n* = 249) were collected in 2017. Briefly, cecal samples were collected after evisceration at the slaughtering line, kept in plastic containers at a temperature of 4–8 °C, and sent to the laboratory for bacteriological analysis within two days.

Upon arrival, samples were inoculated in Heart Infusion Broth with 6% NaCl and incubated at 37 °C for 18 h. The broth cultures were then streaked on the selective medium BBL™ Enterococcosel™ Agar (Becton, Dickinson Company, Wantage, NJ, USA) and incubated under the same conditions. Individual presumptive enterococci colonies were transferred onto Colombia Blood Agar Base (Thermo Scientific™ Oxoid™, Basingstoke, United Kingdom), incubated for 18 to 22 h at 37 °C, and then stored in Tryptone Soy Broth (Thermo Scientific™ Oxoid™, Basingstoke, United Kingdom) with 15% glycerol at −80 °C.

DNA extraction of bacterial isolates followed the boiling lysis procedure, and the concentration and purity of the DNA suspensions were assessed using a NanoDrop™ 2000 Spectrophotometer (Thermo Scientific™, ThermoFisher Scientific, Pittsburgh, PA, USA). Confirmation of presumptive *Enterococcus* colonies was achieved by PCR amplification targeting the 16S rRNA gene as described by Deasy et al. (2000) [79]. 

The identification of five enterococci species (*E. faecium*, *E. faecalis*, *E. hirae*, *E. durans*, and *E. casseliflavus*) was carried out by multiplex PCR using the primer sets designed by Jackson et al. (2004) [80] in optimized thermal cycling conditions (Supplementary Materials Table S1).

Sanger sequencing of the 16S rRNA gene using the primers E1 and E2 described by Jackson et al. (2004) [80] was carried out in isolates not identified by the PCR assays. PCR products were purified using ExoSAP-IT™ (Applied Biosystems™, ThermoFisher Scientific, Pittsburgh, PA, USA) and sequenced at Eurofins Genomics Europe Sequencing GmbH, Konstanz, Germany. The DNA sequences were read with ChromasProTM v2.1.8.0 (Technelysium Pty Ltd, South Brisbane, Australia) and analyzed with the Basic Local Alignment Search Tool (BLAST) [81]. In addition, consensus DNA sequences were generated using BioEdit v7.2.5.0 (Tom Hall Ibis Therapeutics, Carlsbad, CA, USA) sequence alignment editor and FASTA files were further analyzed with BLAST.

Few isolates for which molecular confirmation of species was not conclusive were further identified using the commercially available API^®^ 20 Strep (bioMérieux, Marcy-l’Étoile, France).

### 4.2. Antimicrobial Susceptibility Testing

The antibiotic susceptibility of *E. faecalis* and *E. faecium* isolates was assessed by the agar dilution technique performed according to standard guidelines (CLSI) [82]. The agar dilution plates contained twofold serial dilutions of nine antibiotics (Glentham Life Sciences, Corsham, UK): vancomycin (1–128 µg/mL), teicoplanin (0.5–64 µg/mL), linezolid (0.5–64 µg/mL), tetracycline (1–128 µg/mL), ampicillin (0.5–64 µg/mL), ciprofloxacin (0.12–16 µg/mL), erythromycin (1–128 µg/mL), gentamicin (8–1024 µg/mL) and chloramphenicol (4–128 µg/mL). 

Broth microdilution using Sensititre™ EUVENC plates (Sensititre^®^, Trek Diagnostic Systems, East Grinstead, United Kingdom) was performed in 21 isolates of *E. faecium* (*n* = 6) and *E. faecalis* (*n* = 15).

The antibiotic panels were read using a semi-automatic Sensititre™ Vizion™ Digital MIC Viewing System (ThermoFisher Scientific, Waltham, MA, USA) and the Thermo Scientific™ Sensititre™ SWIN™ Software System (ThermoFisher Scientific, Waltham, MA, USA). Results were assessed with the epidemiological cut-off (ECOFF) values provided by EUCAST. Linezolid clinical breakpoint (MIC > 4 mg/L) from EUCAST was used for *E. faecalis* isolates as no ECOFF is available. Isolates non-susceptible to three or more classes of antibiotics were considered multidrug-resistant.

*Enterococcus faecalis* ATCC 29212 was used as a quality control strain in both procedures.

### 4.3. PCR Screening of Antibiotic Resistance Genes

Molecular screening of vancomycin and linezolid resistance determinants was performed by PCR using primers previously described [14,83,84,85], with cycling conditions and reference strains detailed in Supplementary Table S2. All isolates of *E. faecalis* and *E. faecium* were screened for the *vanA* gene, while the *vanB* gene was searched in isolates that exhibited vancomycin MIC ≥ 2 µg/mL. Moreover, isolates with linezolid and chloramphenicol MIC ≥ 2 µg/mL and MIC ≥ 32 µg/mL, respectively, were subjected to a PCR assay targeting the *optrA* and *cfr* genes.

### 4.4. Whole-Genome Sequencing

Whole-genome sequencing (WGS) was conducted on five *E. faecalis* and two *E. faecium* isolates harboring the *optrA* gene, and on two daptomycin-susceptible *E. faecalis* strains all recovered from swine. DNA extraction was performed using PureLink^®^ Genomic DNA mini kit, Gram-positive bacterial cell lysate protocol (Invitrogen, Carlsbad, CA, USA) according to the manufacturer’s instructions. Library preparation and DNA sequencing were performed by Novogene Europe, Cambridge, UK, using Illumina HiSeq sequencing technology (NovaSeq 6000 S2 PE150 XP sequencing mode).

Sequencing data quality was assessed by FastQC [86] and Trimmomatic v0.27 [87] was used with default settings to remove low-quality data and adapter sequences. Pre-processed reads were assembled with SPAdes 3.12.0 [88] and the assembly stats were calculated using QUAST-5.0.2 [89]. Contigs with sizes lower than 500 bp were removed and nucleotide sequences were analyzed using tools available on the Center for Genomic Epidemiology (CGE) website [90].

Bioinformatics tools ResFinder v4.0 (90% threshold for %ID/60% minimum length) [90,91], PlasmidFinder (95% threshold for %ID) [92,93] and VirulenceFinder v2.0 (90% threshold for %ID/60% minimum length) [94] were used to screen for acquired antimicrobial resistance genes, plasmid sequences and virulence genes, respectively. Multi-locus sequence types (MLSTs) were assigned using MLST version 2.0 [95] on CGE [96] and the MLST database on PubMLST [61].

The Basic Local Alignment Search Tool (BLAST) [81] was used to determine *optrA* and *poxtA* variants.

Linezolid resistant isolates were screened for mutations in the ribosomal proteins L3 and L4 by aligning the respective amino acid sequences with those of reference strains *E. faecalis* ATCC 29212 (GenBank accession number CP008816.1) and *E. faecium* ATCC 8459 (GenBank accession number CP004063.1) using the Molecular Evolutionary Genetics Analysis software (MEGAX) [97].

To identify the genetic platform of the *optrA* gene, contigs containing this gene were annotated using Prokka v1.14.6 [98], followed by analysis with Artemis [99], EasyFig v2.2.5 [100], and BLAST.

### 4.5. Statistical Analysis

Fisher’s exact test was used with a 95% confidence level to analyze statistical data on Microsoft Excel to verify the association between animal species and antimicrobial susceptibility.

### 4.6. Accession Numbers

Raw sequence data obtained from all the sequenced isolates were submitted to the European Nucleotide Archive (ENA) under study accession numbers: ERS6142029, ERS11708758, ERS6142031, ERS11708754, ERS11708755, ERS11708756, ERS11708757 ERS11708759 and ERS11708760.

## 5. Conclusions

Our results underline the impact of the administration of certain antibiotic classes and differences in husbandry and antibiotic use practices in the gut microbiome of clinically healthy cattle and pigs.

The findings of *Enterococcus* spp. strains from pigs resistant to last-resort antimicrobials linezolid and daptomycin are worrying, posing a risk to human health. This is because enterococci in pigs can serve as reservoirs for resistance genes. Moreover, the co-occurrence of resistance mechanisms may perpetuate the emergence and spread of *optrA* and *poxtA* under the selective pressure of amphenicols, even in the absence of oxazolidines usage. Besides clinically relevant lineages like *E. faecalis* ST16 and *E. faecium* ST22, several other lineages, including new STs, were found, suggesting a high diversity among enterococci circulating in pig production in Portugal.

In addition, the emergence of daptomycin non-susceptible *E. faecalis* strains should be carefully monitored, and further research to assess the molecular basis of daptomycin resistance should be performed.

Surveillance programs and research studies to investigate the prevalence and molecular mechanisms of antibiotic resistance in the commensal flora of farm animals are of utmost importance to establishing the risks of the transmission of antibiotic-resistant enterococci from animals to humans and vice versa from a One Health Perspective.

## Figures and Tables

**Table 1:** Antimicrobial susceptibility of *E. faecalis* and *E. faecium* isolates (*n* = 132) using the agar dilution technique.

Antimicrobial	Criteria ^(a)^	*E. faecalis* (*n* = 84)			*E. faecium* (*n* = 48)		
		(T)ECOFF ^(b)^	Cattle (*n* = 18)	Pigs (*n* = 66)	(T)ECOFF ^(b)^	Cattle (*n* = 12)	Pigs (*n* = 36)
Vancomycin	MIC_50_	4	≤1	≤1	4	≤1	≤1
	MIC_90_		4	2		≤1	≤1
	% DS		0	0		0	0
Teicoplanin	MIC_50_	2	≤0.5	≤0.5	2	≤0.5	≤0.5
	MIC_90_		≤0.5	≤0.5		1	≤0.5
	% DS		0	0		0	0
Tetracycline	MIC_50_	4	≤1	128	4	16	128
	MIC_90_		64	128		128	>128
	% DS		44 ^(c)^	98 ^(c)^		58	78
Ciprofloxacin	MIC_50_	4	1	1	8	2	1
	MIC_90_		2	4		4	2
	% DS		0	9		0	0
Erythromycin	MIC_50_	4	≤1	>128	4	≤1	>128
	MIC_90_		>128	>128		2	>128
	% DS		17 ^(c)^	86 ^(c)^		0 ^(c)^	58 ^(c)^
Linezolid	MIC_50_	ND	2	1	4	2	2
	MIC_90_		2	2		2	2
	% DS		-	-		0	0
Gentamicin	MIC_50_	64	≤8	≤8	32	≤8	≤8
	MIC_90_		≤8	128		≤8	≤8
	% DS		0	11		0	0
Ampicillin	MIC_50_	4	≤0.5	1	8	1	1
	MIC_90_		2	2		1	8
	% DS		0	0		0	6
Chloramphenicol	MIC_50_	32	≤4	8	32	≤4	≤4
	MIC_90_		8	64		≤4	16
	% DS		0 ^(c)^	27 ^(c)^		8	3

DS, Decreased susceptibility according to the epidemiological breakpoints; ND, Not determined. ^(a)^ MIC50/90 (µg /mL), % resistance (R) is based on the summed isolate numbers; ^(b)^ Tentative epidemiological cut-off values established by EUCAST; ^(c)^ *p*-value ≤ 0.05.

**Table 2:** OptrA variants and linezolid MICs detected among *E. faecium* (*n* = 2) and *E. faecalis* (*n* = 5) isolates from pigs.

Strain	Species	Linezolid Susceptibility		OptrA Variant			
		EUVENC MIC (µg/mL)	Interpretation ^(a)^	Morroni et al. [32]	Freitas et al. [33]	Almeida et al. [34]	Amino Acid Substitutions
INIAV173	*E. faecium*	4	S	DVD ^(b)^	OptrA_28 ^(b)^	V12	Y176D, A350V, G393D
INIAV004	*E. faecium*	8	R	WT	OptrA_1	V19	None
INIAV168	*E. faecalis*	8	R	DP	OptrA_8	V22	Y176D, T481P
INIAV169	*E. faecalis*	4	S	EDD	OptrA_7	V34	K3E, Y176D, G393D
INIAV170	*E. faecalis*	4	S	EDD	OptrA_7	V34	K3E, Y176D, G393D
INIAV171	*E. faecalis*	8	R	DP	OptrA_8	V22	Y176D, T481P
INIAV174	*E. faecalis*	2	S	EDD	OptrA_7	V34	K3E, Y176D, G393D

MIC, Minimum Inhibitory Concentration; S, Susceptible; R, Resistant. ^(a)^ According to clinical breakpoints provided by EUCAST. ^(b)^ Tentative variant name following the criteria set in the references abovementioned

## Data Availability

The data that support the findings of this study are available within the article.

## Supplementary Materials

The following supporting information can be downloaded at: https://www.mdpi.com/article/10.3390/antibiotics11050615/s1, Table S1: Description of primer sets, annealing temperature, and DNA from control strains used for the molecular species identification of *Enterococcus* spp. Table S2: Description of the primer sets, annealing temperatures and DNA from control strains used for the molecular detection of *vanA*, *vanB*, *optrA*, and *cfr* are available.

## Appendix A

**Table A1:** Genomic characterization of *Enterococcus faecium* (*n* = 2) and *Enterococcus faecalis* (*n* = 7) strains from pig cecal samples.

Isolate	Species	Sample Source	Geographic Location	MLST	EUVENC MDR Profile	Acquired Antimicrobial Resistance Genes and Mutations	Plasmid Replicons	Virulence Genes	Accession Number
INIAV004	*E. faecium*	Swine	39.2333, −8.68333	ST22	TET-ERY-LZD-CLO	***poxtA***, ***optrA***, *fex*(B), *tet*(M), *tet*(L), *erm*(A), *pbp5* (T172A), *pbp5* (L177I), *pbp5* (A216S), *pbp5* (P667S), *pbp5* (D204G), *pbp5* (K144Q), *pbp5* (R34Q), *pbp5* (S27G), *pbp5* (E100Q), *pbp5* (A499T), *pbp5* (G66E), *pbp5* (T324A), *pbp5* (A68T), *pbp5* (V24A), *pbp5* (N496K), *pbp5* (E525D), *pbp5* (E85D)	*rep29*, *rep33*, *repUS43*, *rep1*, *rep2*, *repUS15*	***acm*** **, *efaAfm***	ERS6142029
INIAV173	*E. faecium*	Swine	38.9167, −9.2667	ST2138	TET-ERY-CLO	***poxtA***, ***optrA***, *fex*(B), *erm*(A), *ant(9)-Ia;*	*rep1*; *rep11c*; *rep18b*; *rep29*; *repUS15*; *repUS43*	*acm*; *efaAfm*	ERS11708758
INIAV005	*E. faecalis*	Swine	38.9485, −9.1967	ST93	TET-ERY- DAP	*aac(6′)-aph(2*″), *tet*(M), *tet*(L), *erm*(B)	*rep9a*	*elrA*, *srtA*, *ace*, *cCf10*, *cOB1*, *cad*, *camE*, *ebpA*, *ebpC*, *efaAfs*, *hylA*, *tpx*,	ERS6142031
INIAV168	*E. faecalis*	Swine	41.4124, −8.5206	ST207	TET -ERY-LZD-CLO	***optrA***; *fex*(A), *tet*(M), *tet*(L), *erm*(A), *erm*(B), *clpL*	*repUS1*; *rep9b*; *repUS43*	*elrA*; *srtA*; *ace*; *agg*; *cCF10*; *cOB1*; *cad*; *camE*; *ebpA*; *ebpC*; *efaAfs*; *fsrB*; *gelE*; *hylB*; *tpx*	ERS11708754
INIAV169	*E. faecalis*	Swine	41.4124, −8.5206	ST474	TET-ERY-CIP-GEN-CLO	***optrA***, *fex*(A), *cat*, *aac(6′)-aph(2*″), *aph(3′)-III*, *tet*(M), *tet*(L), *erm*(A), *erm*(B), *dfrG*, *lnu(B)*; *gyrA* (E87G), *parC* (S80I), *lsa*(E)	*rep9a*; *repUS43*	*elrA*; *srtA*; *ace*; *cCF10*; *cOB1*; *cad*; *camE*; *ebpA*; *ebpC*; *efaAfs*; *fsrB*; *gelE*; *hylA*; *hylB*; *tpx*	ERS11708755
INIAV170	*E. faecalis*	Swine	38.7058, −8.97462	ST474	TET-ERY-CIP-GEN-CLO	***optrA***, *fex*(A), *cat*, *aac(6*′*)-aph(2*″), *aph(3*′*)-III*, *tet*(M), *tet*(L), *erm*(A), *erm*(B), *dfrG*, *gyrA* (E87G), *parC* (S80I), *lnu*(B), *lsa*(E)	*rep9a*; *repUS43*	*elrA*; *srtA*; *ace*; *cCF10*; *cOB1*; *cad*; *camE*; *ebpA*; *ebpC*; *efaAfs*; *fsrB*; *gelE*; *hylA*; *hylB*; *tpx*	ERS11708756
INIAV171	*E. faecalis*	Swine	39.4598, −8.6671	ST16	TET-ERY-LZD-CLO	***optrA***, *fex*(A), *cat*, *aac(6′)-aph(2*″), *aph(3′)-III*, *str*, *tet*(M), *erm*(A), *erm*(B), *str*, *lnu*(B), *lsa*(E)	*rep6*; *rep9b*; *repUS43*	*elrA*; *srtA*; *ace*; *agg*; *cCF10*; *cOB1*; *cad*; *camE*; *cylA*; *cylL*; *cylM*; *ebpA*; *ebpC*; *efaAfs*; *hylA*; *tpx*	ERS11708757
INIAV174	*E. faecalis*	Swine	38.9167, −9.2667	ST1178	TET-ERY-CIP-CLO	***optrA***, *cat*, *ant(9)-Ia*, *tet*(M), *tet*(L), *erm*(A), *erm*(B), *gyrA* (E87G), *parC* (S80I)	*rep9a*; *repUS43*	*elrA*; *srtA*; *ace*; *cCF10*; *cOB1*; *cad*; *camE*; *ebpA*; *ebpC*; *efaAfs*; *fsrB*; *gelE*; *hylA*; *hylB*; *tpx*	ERS11708759
INIAV175	*E. faecalis*	Swine	39.4598, −8.6671	ST58	TET-ERY-DAP	*tet* (M), *erm*(B), *ant(6)-Ia*, *dfrG*, *lnu*(B), *lsa*(E)	*repUS43*	*elrA*; *srtA*; *ace*; *cCF10*; *cOB1*; *cad*; *camE*; *ebpA*; *ebpC*; *efaAfs*; *fsrB*; *gelE*; *hylA*; *tpx*	ERS11708760

MLST, Multilocus Sequence Type; MDR, Multidrug resistance; TET, Tetracycline; ERY, Erythromycin; CIP, Ciprofloxacin; GEN, Gentamicin, LZD: Linezolid; CLO, Chloramphenicol; Linezolid resistance genes are highlighted in bold.
