# Supplementary material for: Antimicrobial Susceptibility of Enterococcus Isolates from Cattle and Pigs in Portugal: Linezolid Resistance Genes optrA and poxtA
Source: Antibiotics (Basel). 2022 May 3;11(5):615. doi: 10.3390/antibiotics11050615 (PMC9137492; doi:10.3390/antibiotics11050615)
Supplement: Supplementary file 1 [file antibiotics-11-00615-s001.zip › antibiotics-1694947-supplementary.pdf]

Supplemantary Materials

Table S1. Description of primer sets, annealing temperature and DNA from control strains used for the molecular species identification of *Enterococcus* spp.

Target taxon	Target genes	Primers	Sequence (5′-3′)	Amplicon size	Annealing temperature	PCR-Positive control strain	Ref
Enterococcus	16S rRNA	E1	TCAACCGGGGAGGGT	733 bp	55ºC	ATCC 29212	79
		E2	ATTACTAGCGATTCCGG				
E. faecalis	*sodA*	FL1	ACTTATGTGACTAACTTAACC	360 bp	55ºC	ATCC 29212	80
		FL2	TAATGGTGAATCTTGGTTTGG				
E. faecium	*sodA*	FM1	GAAAAAACAATAGAAGAATTAT	215 bp	55ºC	INIAV 127P	80
		FM2	TGCTTTTTTGAATTCTTCTTTA				
E. hirae	*sodA*	HI1	CTTTCTGATATGGATGCTGTC	187 bp	48ºC	INIAV 27P	80
		HI2	TAAATTCTTCCTTAAATGTTG				
E. casseliflavus	*sodA*	CA1	TCCTGAATTAGGTGAAAAAAC	288 bp	50ºC	AUT 14A	80
		CA2	GCTAGTTTACCGTCTTTAACG				
E. durans	*sodA*	DU1	CCTACTGATATTAAGACAGCG	295 bp	50ºC	AUT 49B	80
		DU2	TAATCCTAAGATAGGTGTTTG				

Table S2. Description of the primer sets, annealing temperatures and DNA from control strains used for the molecular detection of *vanA, vanB, optrA, and cfr*

Target genes	Primers	Sequence (5′-3′)	Amplicon size	Annealing temperature	PCR-positive control strains	Ref.
vanA	VanA1	AAAGTGCGAAAAACCTTGC	535 bp	54˚C	*E. faecium* BM4	83
	VanA2	AACAACTTACGCGGCACT				
vanB	EB3	ACGGAATGGGAAGCCGA	647 bp	54˚C	*E. faecalis* V583	84
	EB4	TGCACCCGATTTCGTTC				
optrA	optrA_F	AGGTGGTCAGCGAACTAA	1395 bp	54˚C	*E. faecalis* INIAV 100P	14
	optrA_R	ATCAACTGTTCCCATTCA				
cfr	cfr_F	TGAAGTATAAAGCAGGTTGGGGT	746 bp	48˚C	*Staphylococcus aureus* INIAV001	85
	cfr_R	ACCATATAATTGACCACAGC GC				
